# Uniting Superhydrophobic, Superoleophobic and Lubricant Infused Slippery Behavior on Copper Oxide Nano-structured Substrates

**DOI:** 10.1038/srep35524

**Published:** 2016-10-18

**Authors:** Sanjeev Kumar Ujjain, Pritam Kumar Roy, Sumana Kumar, Subhash Singha, Krishnacharya Khare

**Affiliations:** 1 Department of Physics, Indian Institute of Technology Kanpur, Kanpur - 208016, India

## Abstract

Alloys, specifically steel, are considered as the workhorse of our society and are inimitable engineering materials in the field of infrastructure, industry and possesses significant applications in our daily life. However, creating a robust synthetic metallic surface that repels various liquids has remained extremely challenging. The wettability of a solid surface is known to be governed by its geometric nano-/micro structure and the chemical composition. Here, we are demonstrating a facile and economical way to generate copper oxide micro-nano structures with spherical (0D), needle (1D) and hierarchical cauliflower (3D) morphologies on galvanized steel substrates using a simple chemical bath deposition method. These nano/micro textured steel surfaces, on subsequent coating of a low surface energy material display excellent superhydrophobic, superoleophobic and slippery behavior. Polydimethylsiloxane coated textured surfaces illustrate superhydrophobicity with water contact angle about 160°(2) and critical sliding angle ~2°. When functionalized with low-surface energy perfluoroalkylsilane, these surfaces display high repellency for low surface tension oils as well as hydrocarbons. Among them, the hierarchical cauliflower morphology exhibits re-entrant structure thereby showing the best superoleophobicity with contact angle 149° for dodecane. Once infused with a lubricant like silicone oil, they show excellent slippery behavior with low contact angle hysteresis (~ 2°) for water drops.

Past decade has attracted tremendous scientific interest in superhydrophobic surfaces because of their potential applications in many fields, such as transport of microdroplets, biochemical separation, drug delivery, tissue engineering, anticorrosion, self-cleaning, drag-reduction coating and microfluidic lab-on-chip devices^1^,^2^,^3^,^4^,^5^,^6^,^7^,^8^,^9^,^10^,^11^,^12^. Inspired by the investigation on lotus leaf effect, synthetic superhydrophobic surfaces are fabricated on a variety of substrates including metals^13^, glass^14^, polymers^15^ and fabrics^16^ by combining hierarchical nano- and microstructures along with low surface energy fluorinated molecules, resulting in very high water contact angle (CA > 150°) and a very low sliding angle (SA < 10°)^17^,^18^. In addition to repelling water, superoleophobic surfaces repel organic liquids (oils/hydrocarbons) with low surface tensions and thus create surfaces which are resistant to organic contaminations^19^. However, in contrast to superhydrophobicity, achieving superoleophobicity entails a second essential feature related to a very specific surface morphology; i.e., re-entrant/overhanging surface features^19^,^20^. Generally, fabrication of surfaces with diminished wettability relies on roughness of textured surface since all non-textured (smooth) surfaces, regardless of their chemical compositions, are intrinsically oleophilic. Young’s relation for determining wettability (contact angle *θ*_*Y*_) of a liquid on smooth surface is given by:


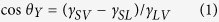


Here *γ* denotes the interfacial surface tension, and *S*, *L*, and *V* stands for solid, liquid and vapor phase, respectively. Non-textured surfaces, if modified with low surface energy fluorosilane molecules (one with lowest surface energy reported is *γ*_*SV*_ = 6 mJ/m^2^)^21^ result in contact angle (*θ*_*Y*_) < 90° for oils. For instance, hexadecane (surface tension *γ*_*LV*_  = 27.6 mJ/m^2^) showed *θ*_*Y*_ ~ 80° while for water (surface tension *γ*_*LV*_ = 72.1 mJ/m^2^) *θ*_*Y *_~ 120°^22^. On surfaces with small roughness, contact angle is determined by Wenzel’s relation as 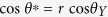 where *r* is the roughness parameter defined as the ratio of actual surface area to projected surface area^23^. In contrast, surfaces with large roughness modified with fluorinated silanes demonstrated *θ* > 150° for oils and *θ* > 160° for water^20^ with very low roll-off angle (*ω* ≤ 2°) as the liquid droplets can be stabbed on the top of the roughness asperities due to pockets of air trapped underneath^24^,^25^,^26^,^27^,^28^,^29^,^30^,^31^,^32^,^33^,^34^,^35^,^36^, as discussed by Cassie-Baxter relation:


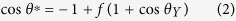


where θ^*^ is defined as apparent contact angle on textured (rough) surface and *f* represents the area fraction of solid-liquid interface. Although this approach is promising, it suffers from inherent limitations related to irreversible defects arising during fabrication and mechanical damage which enhance pinning effect in the superhydrophobic surface and deteriorate liquid mobility^37^. In order to overcome these limitations, taking inspiration from Nepenthes pitcher plants^38^, stable, defect-free and inert ‘slippery’ interface have been developed by lubricating liquid-infused porous surface in which micro, nanostructures locked the infused lubricant in place^37^,^39^,^40^,^41^,^42^,^43^,^44^,^45^,^46^,^47^,^48^. Lubricating fluid is intrinsically smooth and defect-free and can afford instantaneous self-repair by wicking into dent sites in the underlying substrate^37^,^38^. Lubricating fluid may not have the ability to self-repair in presence of ice as shown by Rykaczewski *et al*.^49^. Recently, Anand *et al*. has demonstrated reduced pinning of the condensate droplets by a hierarchical micro-nano textured surface by impregnating with an appropriate lubricant^50^ while, Li *et al*. suggested self cleaning properties of hydrophobic liquid-infused porous poly(butyl methacrylate-co-ethylene dimethacrylate) surface^51^.

Therefore, it would be worthwhile to notice that combination of the nano/micro, hierarchical and re-entrant structures along with well-matched solid and liquid surface energy is the most crucial parameter to create highly stable superhydrophobic, superoleophobic and slippery surfaces but making such robust textured roughness is challenging^37^. Although the current state-of-the-art have generated efficient surfaces with precise control of nano and micro structures, however reported fabrication processes for the creation of surface roughness involving lithographic means^37^,^52^, micro-fabrication^53^,^54^,^55^, self assembly^56^,^57^, or by the use of polyelectrolyte multilayers (PEMs) assembled by the layer-by-layer technique^58^, sol-gel methods^59^, spin-coating^60^, electrochemical deposition which requires a conductive substrate^61^, and polymer imprinting^2^,^62^,^63^,^64^,^65^,^66^,^67^ are sophisticated, costly and difficult to be implemented on a large scale. The complications are mostly associated with the manufacturing of a robust hierarchical structure with re-entrant and convex morphology which is the key for superoleophobicity.

Steel, considered to be the workhorse of our society as the most essential engineering material in field of construction, food, petrochemical, maritime and aviation industries^68^. Its broad applications can be further augmented by making it super-repellent for water/oil, especially for industries where metal−fluid contact is common. The super-repellency with such fluids provides resistance to fouling/corrosion properties which subsequently increases its life-time^69^.

In this work, we have utilized relatively simple chemical bath deposition (CBD) technique for synthesizing four different morphologies of copper oxide (CuO) nano-/micro and hierarchical structures on steel substrates. The CBD method involves very simple instrumentation facility and can be used to fabricate a single crystalline material such as metal oxides and hydroxides on a wide variety of substrates, because in thermodynamic equilibrium conditions each metal complex in the precursor solution is singly deposited on the substrate surface^70^. Furthermore, these CuO nano-/micro textured steel substrates were coated with polymethylsiloxane (PDMS) by dip coating, resulting in highly robust superhydrophobic surfaces. The effect of micro-/nano structures on superhydrophobic, superoleophobic and lubricant based slippery behavior have been studied. Moreover, the fabrication conditions involved in the whole process are mild (low temperature, dilute solutions and air atmosphere) and fairly easy, required no sophisticated instrument.

## Experimental Section

### Materials

Poly(dimethylsiloxane) (PDMS) pre-polymer (Sylgard 184A) and thermal curing agent (Sylgard 184B) were purchased from Dow Corning Corp. 1H,1H,2H,2H-perfluorooctyltriethoxy silane (C_14_H_19_F_13_O_3_Si) (PTES) was purchased from Alfa Aesar. Silicone oil (*η* = 350 cSt), copper sulphate (CuSO_4_), L-ascorbic acid, sodium hydroxide (NaOH) and sodium borohydride (NaBH_4_) were of analytical grade and purchased from Loba Chemie. Sodium dodecyl sulphate (SDS), ethanol and toluene were procured from Merck. Galvanized steel (GI) substrates (Tata Steel, India) (4 cm × 2 cm × 0.2 cm) were ultrasonically cleaned in acetone and then rinsed with de-ionized water sequentially before use.

### Characterization

The surface morphologies of the fabricated copper oxide particles on different substrates and the corresponding superhydrophobic PDMS films coated samples were investigated using field emission scanning electron microscopy (FESEM: JEOL, JXA-8230) at an accelerating voltage of 10 kV and RMS surface roughness was determined using atomic force microscope (AFM) (Park system XE-70) in a tapping mode in the range of scanning area of 3 μm × 3 μm. Powder X-ray diffraction (XRD) measurements of copper oxide nanoparticles grown on steel substrates were performed with a X’Pert Pro MPD X-Ray Diffractometer. Static contact angle (CA) (*θ*_*S*_), Advancing contact angle (*θ*_*A*_) and Receding contact angle (*θ*_*R*_) were measured using Milli-Q water at room temperature with a Contact angle goniometer (OCA-35, DataPhysics, Germany). *θ*_*S*_ was measures using Laplace-Young fitting model of a 2 μl water droplet placed on the horizontal glass substrate. *θ*_*A*_ and *θ*_*R*_ were measured by adding and then withdrawing 2 μl of water drop respectively. The contact angle hysteresis (*θ*_*A*_ − *θ*_*R*_) (CAH) was obtained using the circle fitting method. Static tilt angle (α) was obtained by observing the minimum tilt angle required to move the water drop (10 μl) from horizontal surface. The velocity of the drop (10 μl) was measured by recording the movement of water drops on a tilted surface (α = 12°) and then calculating the distance travelled per unit time. All *θ* and α value are in the range of ±2°. All the measurements were performed in duplicate on a given substrate and two such substrates for each composition. The values mentioned are average of these measurements.

### Synthesis of copper oxide nanoparticles

Morphology controlled growth of copper oxide nanoparticles film on Fe substrate in aqueous solvent was typically processed by bath deposition method. Equal volume of two aqueous sodium dodecyl sulfate (SDS) solutions (250 mg in 20 ml DI), one containing cupric sulphate (CuSO_4_.5H_2_O) (10 mM to 100 mM solution in DI water) and the other containing L-ascorbic acid (20 mM to 200 mM) were mixed by magnetic stirrer and temperature was kept constant at 70 °C. Color change occurred in the aqueous phase from blue to pale green and finally the solution turned colorless. The pH of the solution was adjusted to 11 using NaOH solution. On addition of NaOH solution, the colorless solution changed to yellow, reddish orange and reddish brown respectively, depending on the concentration of metal precursor solutions. The cleaned steel substrates of dimension (4 cm × 2 cm) were immersed in the above solution. After stirring for 10 min, 10 ml of (100 mM to 1M) NaBH_4 _aqueous solution was drop wise added into the reaction vessel. On addition, all the solutions gradually became reddish black. The reaction mixture was further stirred overnight in ambient atmosphere at 70 °C, to allow the reaction to complete. Thin film of CuO nanoparticles formed on steel substrates was taken out and washed with ethanol and DI water to remove surfactant. The films were dried in air at 60 °C and used for further studies. For synthesizing CuO nanoparticles on PDMS film, glass and steel mesh substrates, highest precursor concentration (i.e. 100 mM CuSO_4_) was used.

### Preparation of PDMS coated superhydrophobic copper oxide textured steel

Copper oxide nanoparticles textured steel surfaces were found to be superhydrophilic. In order to make them superhydrophobic, PDMS was used as a low surface energy material for the nanostructured substrates. PDMS pre-polymer and thermal curing agent (Sylgard®184, Dow Corning) were mixed in 2:1 ratio and diluted in 200 ml toluene. The nano-patterned surfaces were dip-coated in the solution (Apex Instrument, Model Xdip-SV1) with 200 mm/min lifting speed which were subsequently annealed at 110 °C for 30 min. Since it was not possible to measure the PDMS thickness on the nano-patterned substrates, a flat CuO surface was also used as control substrate and coated with PDMS using same parameters which gave PDMS thickness of 32 (±6) nm as measured using an Atomic Force Microscope (AFM). Therefore we can assume that PDMS thickness on the nano-patterned substrates would also be in the range from 20–40 nm.

### Surface modification by perfluoroalkylsilane

The PDMS coated copper oxide textured steel samples were exposed to a UV Ozone cleaner for 10 min to oxidize the PDMS surface and generate oxygen functionalities^71^. These functionalities act as anchor sites for perfluoroalkylsilane grafting. In a typical process, the oxidized samples were placed in a vacuum chamber and 20 μl of 1H,1H,2H,2H-perfluorooctyltriethoxysilane (C_14_H_19_F_13_O_3_Si) (PTES), deposited on a glass slide was placed at a sufficient minimum distance from samples. Under low pressure, PTES vapor was deposited on samples for 20 min.

### Silicone oil infused slippery surface

Slippery surfaces based on PDMS coated copper oxide textured steel surfaces infused with Silicone oil were prepared by immersing the steel samples in silicone oil for 2 min and then pulled out with a speed of 200 mm/min. The porous PDMS absorbs silicone oil and excess oil was removed by gravity drainage by hanging samples for 30 min. After gravity drainage, the substrates were spin coated at 500 rpm for 30 sec to provide uniformity in the oil film thickness. It is difficult to define the lubricating film thickness on nano-patterned substrates since most of the lubricant impregnates into the nano-pores formed by CuO nanostructures. For Set B samples, thickness of CuO nanostructure film is 5.44 (±0.21) *μ*m, therefore we expect that the thickness of the lubricating film on the nano-patterned substrates would be slightly more than the thickness of the nano-pattern itself. Along with the nano-patterned substrates, a control sample (flat CuO surface with thin PDMS layer) was also coated with silicone oil which resulted in oil film thickness of 4.3 (±0.1) *μ*m calculated by weight difference method. Based on these results, we expect the lubricating film thickness on the nano-patterned substrates in the range 5–6 *μ*m.

## Results and Discussion

### Structure, morphology and roughness

Morphology controlled synthesis of nano-crystals with well defined shape and uniform size has been achieved by several methods involving conventional solid state process and wet synthetic routes, such as hydrolysis, pyrolysis, precipitation, and hydrothermal/solvothermal. Among all these methods, the solution assisted synthesis by chemical bath deposition (CBD) of the precursor may be the most facile and effective approach to develop nano-crystals at relatively low temperatures, which is exempted from post calcination.

**Figure 1:**
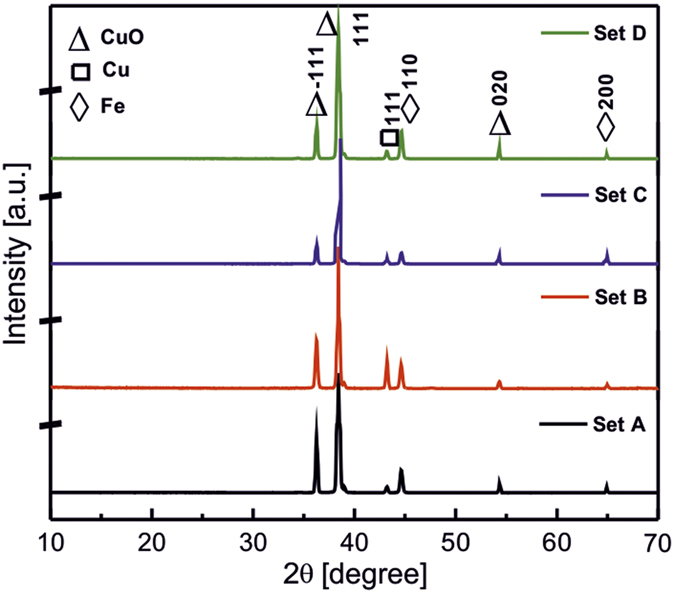
XRD pattern of CuO nanoparticles on steel substrates obtained by the chemical bath deposition method using different concentrations (Set A) 10 mM, (Set B) 20 mM, (Set C) 50 mM and (Set D) 100 mM aqueous CuSO_4_ solution in the presence of the reducing agents ascorbic acid and sodium borohydride respectively at pH 11.

Besides this, CBD exhibits considerable influence of metal salt precursor (CuSO_4_) concentration on the final structure and morphology of the as-prepared CuO patterns on steel substrate. All obtained CuO samples are of Base-centered monoclinic structure, space group: C2/c(15) (JCPDS Card No. 001-1117, a = 4.653 Å, b = 3.41 Å, c = 5.108 Å, b = 99.48°). The XRD pattern shown in Fig. 1 exhibits peak at 2*θ*: 35.74, 38.95, 53.88 corresponding to Miller indices (−111), (111) and (020), confirming CuO. Very diminished peak at 43.3° corresponds to (111) plane of Cu (JCPDS Card No. 01-070-3039). Presence of two characteristic peaks for Fe at 2*θ*: 44.65 and 64.98 corresponding to (110) and (200) Miller indices confirmed the presence of Fe substrate underneath CuO.

**Figure 2:**
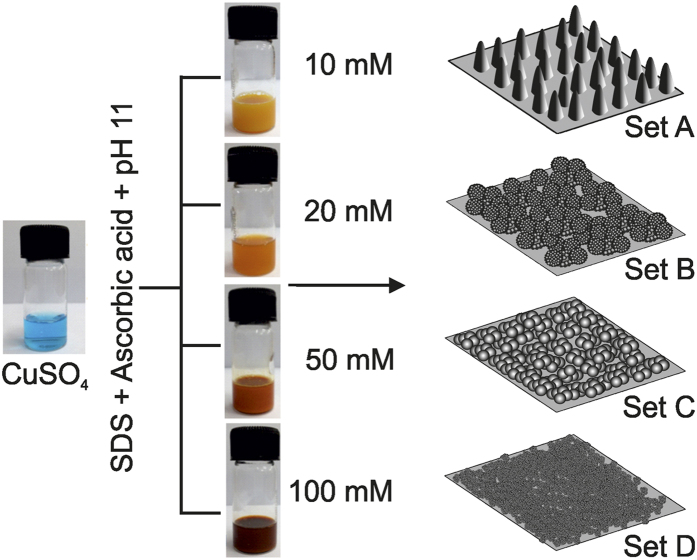
Schematic illustrating precursor concentration dependent CuO nano-/micro particles growth on steel substrates by chemical bath deposition method.

**Figure 3:**
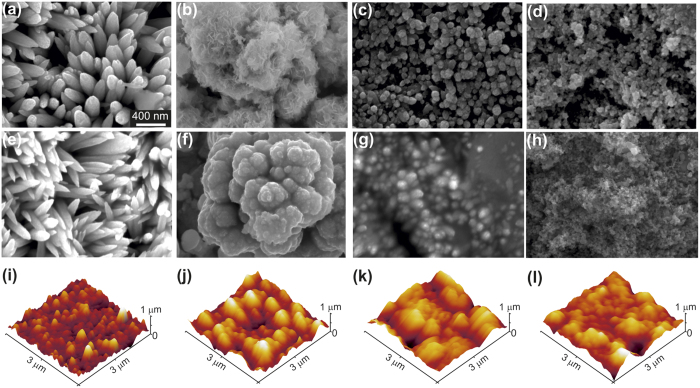
SEM and AFM images of CuO nanostructures obtained by CBD process by varying the concentration of CuSO_4_ precursor solution. (**a**), (**e**,**i**) Nano-needle (10 mM), (**b**), (**f**,**j**) Hierarchical cauliflower (20 mM), (**c**), (**g**,**k**) nanosphere (50 mM) and (**d**), (**h**,**l**) nanosphere cluster (100 mM).

The summarized reaction conditions and morphologies are illustrated in Fig. 2 and the representative SEM and AFM images are shown in Fig. 3. The precursor solution for the crystallization of CuO was prepared using de-ionized water, CuSO_4_, surfactant SDS, L-ascorbic acid, NaOH and NaBH_4_ solution in the order depicted above. Depending on the concentration of aqueous SDS CuSO_4_ solution (blue) from 10 to 100 mM, they demonstrate change in color from yellow to reddish wine on addition of L-ascorbic acid at constant pH 11. After adding reducing agent NaBH_4_, the crystallization of CuO in different morphology took place. The increase in concentration of CuSO_4_ induced a reduction in crystallite size probably due to enhancement of the nucleation rate which enhanced growth kinetics of nanocrystals. SEM images clearly demonstrate the delicate morphology control that can be achieved by adjusting the concentration of precursor solution. 10 mM CuSO_4_ solution results in needle-like morphology with height around 1 μm, (Fig. 3a,e,i) which are almost perpendicular to the substrate. The images show that the needles are very sharp, with tip diameter in the range of tens of nanometer. Upon increasing the CuSO_4_ concentration to 20 mM, hierarchical cauliflower like morphology is obtained with an average diameter of about 0.5 to 2 μm (Fig. 3b,f,j).

Figure 3 (c,g,k) display nano-sphere morphologies with an average diameter 80 nm obtained from 50 mM precursor solution. If concentration is further increased to 100 mM, the CuO nano-spheres get agglomerated resulting in larger clusters (Fig. 3d,h,l). Surface roughness of all the four morphologies calculated from the AFM images are 127.7 nm, 164.5 nm.143.7 nm and 132.9 nm for the nano-needle, hierarchical cauliflower, nanosphere and nanosphere cluster respectively. Thickness of the various CuO nanostructure coating was measured by mechanical profilometer and is shown in Supplementary Figure S1.

In order to reduce surface energy and improve mechanical stability, the hydrophilic CuO nano-patterns were dip coated in dilute PDMS solution followed by curing at 110 °C for 30 min. The resulting CuO nanopatterned surfaces shown in Fig. 3e–h, indicate no change in overall morphology. Here we should note nanoscale roughness in hierarchical cauliflower and nanosphere morphologies is smeared out due to PDMS filling, but we will later show that this does not affect any of the physical behavior of the system.

Mechanical stability of all the fabricated superhydrophobic samples were measured using tape test (ASTM D3359). A cross hatch cutter (Sheen Instruments, model SH750/3) was used to cut the coating followed by peeling with an adhesive tape and this cycle was repeated for 10 times. After each cycle, the surface morphology and wettability were observed to analyze the mechanical stability of the coating. The removed nanoparticles from the CuO nano-needle textured surface without PDMS coating and with PDMS coating were examined by SEM. Dense coverage of nano structures was found on the pealed tape from the surfaces without PDMS coating, while the coated surfaces didn’t reveal much, indicating enhanced stability of nano patterns post PDMS coating. Subsequently, these PDMS coated CuO nano-textured steel surfaces with different morphologies and roughness were investigated for their wetting behavior under different conditions.

### Superhydrophobicity

**Figure 4:**
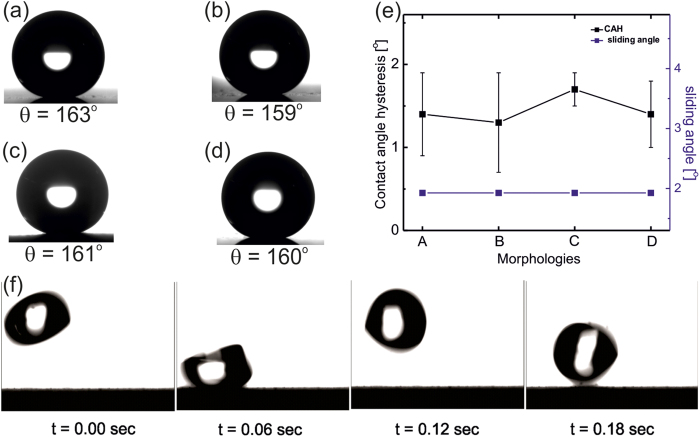
Water contact angle on samples with various morphologies: (**a**) nano-needle, (**b**) hierarchical cauliflower, (**c**) nanospheres and (**d**) nanosphere cluster, (**e**) Contact angle hysteresis and sliding angle plots for different morphologies, (**f**) water drop bouncing off a hierarchical cauliflower based superhydrophobic surface (Set B).

CuO nano-textured PDMS coated steel surface possesses large roughness due to presence of CuO of different morphologies and low surface energy due to PDMS, which is essential to achieve superhydrophobicity^20^. All four CuO nano-textured morphologies after PDMS coating exhibit prominent superhydrophobicity with water contact angles as high as 163° (Fig. 4a–d). The extreme water repellency of each surface also reflects very low contact angle hysteresis (∆*θ* ~ 2°) and very low sliding angles (α ~ 2°) for 2 μl droplet volume (Fig. 4e). The ultra-low contact angle hysteresis (difference between the advancing and receding contact angle of the droplet) and sliding angle (minimum surface tilt on which droplet starts moving) of these surfaces confirm uniformity and lack of pinning sites^37^ which allowed water droplets to easily bounce and roll-off on them (Fig. 4f; see Supplementary Movie S1).

**Figure 5:**
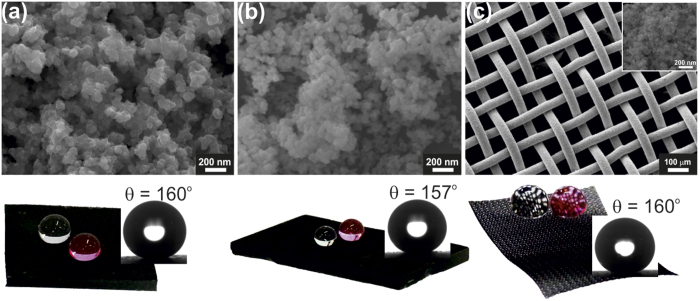
FESEM micrograph of CuO nanoparticles formed on (**a**) PDMS film, (**b**) glass and (**c**) steel mesh (Inset showing high magnification image). Their respective optical photograph with water droplets sitting on the surfaces and contact angle is also shown.

This superhydrophobic nature also protects the surfaces from wide range of contaminants by self-cleaning action which allows water droplets to collect and remove the contaminants from surfaces upon roll off (see Supplementary Movie S2). To demonstrate versatility of CBD based superhydrophobic coatings on variety of substrates, CuO nanopatterns were grown on glass, PDMS sheet and steel mesh followed by PDMS coating. Figure 5a–c shows electron microscope images of CuO nanoparticles based superhydrophobic samples on PDMS, glass and steel mesh along with optical micrographs and water drops. All these substrates showed excellent superhydrophobicity (water contact angle ~160°) and mechanical stability (see Supplementary Figure S2).

### Superoleophobicity

**Figure 6:**
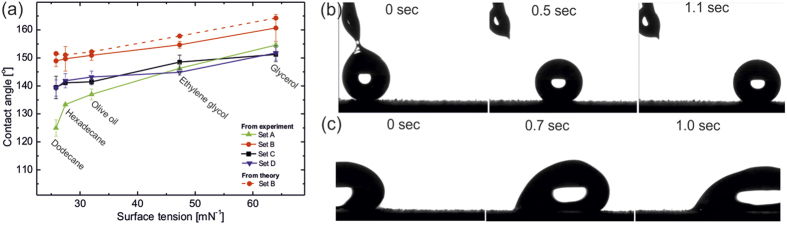
Superoleophobic behavior (**a**) Comparison of contact angle as a function of surface tension of test liquids on various morphologies of perfluoroalkylsilane functionalized PDMS coated CuO on steel substrates (Set A) nano-needle, (Set B) hierarchical cauliflower, (Set C) nanospheres and (Set D) nanosphere cluster. Corresponding theoretical value for Set B is shown with dotted line. Snaps of rolling glycerol (**b**) and dodacane (**c**) drops on hierarchical cauliflower (Set B) textured surface.

To generate repellency towards low surface tension oils and hydrocarbons, the PDMS coated nano-textured surfaces were further functionalized with a low-surface energy perfluoroalkylsilane. Each of these surfaces demonstrates extreme liquid repellency with contact angles 160° to 125° against liquids of surface tension ranging from 64.0 mNm^−1^ (glycerol) to 25.3 mNm^−1^ (dodecane) and depending on different morphologies (Fig. 6a).

The surface with cauliflower morphology having hierarchical nano-/micro structure with highest roughness displays higher contact angles, 160° for glycerol, which decreased to 149° for dodecane (Fig. 6(b,c); see Supplementary Movie S3 & Movie S4), while the nano-needle surface showed inferior repellency for low surface tension liquid, around 125° for dodecane. This is in agreement with previous reports discussing the ideal design parameters for superoleophobic surfaces having re-entrant geometry^20^. Hierarchically structured cauliflower surface, possessing re-entrant structure, can trap higher fraction of air at both the coarser and finer length scales showing extreme superoleophobicity while nano-needle structured surface has one scale of texture. Consequently, the former can support low surface tension liquids in the Cassie state resulting in superoleophobic surface. The substrates possessing a predominantly spherical textures (Set C and Set D) demonstrated intermediate superoleophobic behavior between the hierarchical and one scale textured.

As the cauliflower shape possesses re-entrant nanostructure and demonstrates the highest oleophobicity among the studied textures, we approximated the contact angles for different test liquids using equation (3)^19^,^20^.


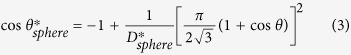


where, θ* is the apparent contact angle on the textured surface and *θ* is the equilibrium contact angle on a smooth surface of the same substrate, given by Young’s equation (1). D* is the spacing ratio defining surface porosity and given by 
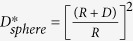
 for spherical textured surface. Here, *R* defines the radius of the sphere and 2*D* is the inter-sphere distance. The theoretical values calculated using the above equation precisely matches the experimental data trend with highest contact angle 164° for glycerol and 151° for dodecane (Fig. 6a). The higher theoretical values for hierarchically textured surface are in agreement with the previous works on designing superoleophobic surfaces^20^.

### Silicone oil infused slippery surfaces (SOIS)

SOIS were designed taking three criteria in consideration: (1) affinity of lubricating liquid with solid surface, i.e. the lubricating liquid must spread completely on the substrate, (2) slipping test liquid must be immiscible with the lubricant and (3) requirement of hydrophobic solid substrate. The first requirement is satisfied by using silicone oil, as it spreads completely on PDMS films. Since silicone oil is known to swell crosslinked PDMS, it is advantageous for our system as the swelling increases its adhesion with PDMS coated substrates. Second criterion was satisfied by taking water as a test liquid which shows immiscibility with silicone oil.

**Figure 7:**
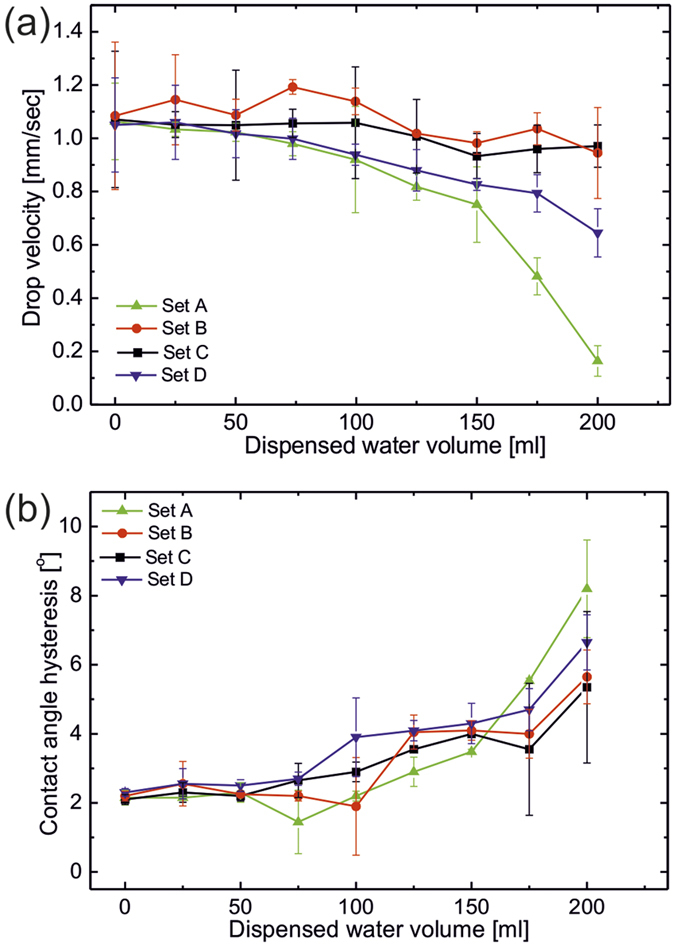
Silicone oil infused slippery behavior for water drops (**a**) Showing 10 μl water drop velocity with volume of water dispensed and (**b**) contact angle hysteresis as a function of dispensed water volume of water dispensed over four different morphologies of Silicone oil infused PDMS coated CuO textured steel surfaces.

Third criterion is fulfilled automatically as PDMS coated substrates are inherently hydrophobic. Satisfying these criteria, we fabricated silicone oil infused nano textured slippery surfaces on steel substrates to slip water droplets upon tilting. Each of these SOIS demonstrates very low contact angle hysteresis (Δθ < 2°) and low critical sliding angle (α < 2°) and good slippery behavior with water drop velocity (≥1.05 mm/sec) for 10 μl droplet volume inclined at 12° (Fig. 7). Slippery behavior of the SOIS is found independent of underlying textured geometry provided sufficient lubricating fluid covers the entire surface. Static water drops deposited on silicone oil coated solid surfaces are cloaked with a thin layer of oil due to positive spreading coefficient of oil on water. Spreading parameter (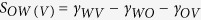, subscript *O*, *W* and *V* represent oil, water and vapour respectively) for our system is 8.5 mN/m^72^. Once these oil cloaked water drops slip from the lubricant coated surface, they slowly remove oil from the surface thus decrease the lubricating layer thickness which affect the velocity of slipping water drop. We studied the effect of CuO nano morphology on the degradation of slippery behavior as a function of slipping water volume (Fig. 7a). It is clear from the figure that set B (hierarchical cauliflower) shows smallest degradation in slip velocity upon slipping water drops. This is due to the fact that the hierarchical cauliflower structures posses re-entrant geometry and lubricating oil trapped in the re-entrant structures are very hard to be removed. Therefore these structures show least degradation upon water flow compared to other structures. On the other hand, degradation on Set A sample is found to be the most due to their needle like structure. This degradation in slippery behavior of SOIS is related to the decreased lubricant thickness, which is also reflected in their increasing contact angle hysteresis (Fig. 7b). Expectedly, set B shows lowest increase in the contact angle hysteresis whereas set A shows the largest due to their underlying morphology. Self-healing ability of the SOIS is also checked by large area physical damage created on silicone oil film using a knife. Silicone oil lubricating film quickly heals the damage and restores the slippery behavior within fraction of seconds by filling the damaged void area by surface capillary action (see Supplementary Movie S5).

## Conclusion

In this article, we have demonstrated a novel and convenient method to synthesize CuO nano-/micro structures with spherical (0D), needle (1D) and hierarchical (3D) cauliflower morphologies on steel substrate using chemical bath deposition (CBD). The change in morphology from 0D to 3D is precursor concentration dependent. Another advantage of this method is that, it is substrate independent and can be formed on polymer film, glass and mesh. On polydimethylsiloxane (PDMS) coating, these nano-/micro textured surfaces formed robust self-cleaning superhydrophobic surfaces with water drops bouncing on them as a consequence of low contact angle hysteresis (Δθ ≤ 2°) and sliding angle (α < 2°). Perfluoroalkylsilane grafting resulted in low surface energy nano-/micro textured substrates showing repellency against various liquids with surface tension ≥25.3 mNm^−1^ (dodecane). The cauliflower morphology textured steel surface outperformed other morphologies in terms of different liquid repellency because of its hierarchical surface. In order to eliminate the constraints related to the self-healing on physical damage, silicone oil infused slippery surfaces (SOIS) were formed on these nano-/micro textured steel substrates. These SOIS demonstrated excellent slippery behavior for water with quick self-healing against physical damage. These results indicate that a suitable nano-/micro textured robust structures for desired application (superhydrophobic/superoleophobic/slippery) can be fabricated very easily on various substrates. We expect this low-cost, fast and convenient method will pave a new way for designing and fabricating robust textured surfaces for numerous applications in research and industrial field.

## Additional Information

**How to cite this article**: Ujjain, S. K. *et al*. Uniting Superhydrophobic, Superoleophobic and Lubricant Infused Slippery Behavior on Copper Oxide Nano-structured Substrates. *Sci. Rep.* **6**, 35524; doi: 10.1038/srep35524 (2016).

## Supplementary Material

Supplementary Information

Supplementary Movie S1

Supplementary Movie S2

Supplementary Movie S3

Supplementary Movie S4

Supplementary Movie S5
