# Supplementary material for: Uniting Superhydrophobic, Superoleophobic and Lubricant Infused Slippery Behavior on Copper Oxide Nano-structured Substrates
Source: Sci Rep. 2016 Oct 18;6:35524. doi: 10.1038/srep35524 (PMC5067640; doi:10.1038/srep35524)
Supplement: Supplementary Information [file srep35524-s1.doc]

**Supporting Information**

**Uniting Superhydrophobic, Superoleophobic and Lubricant Infused Slippery Behavior on Copper Oxide Nano-structured Substrates**

Sanjeev Kumar Ujjain^$^, Pritam Kumar Roy^$^, Sumana Kumar, Subhash Singha and Krishnacharya Khare^*^

Department of Physics, Indian Institute of Technology Kanpur, Kanpur - 208016, India

^*^kcharya@iitk.ac.in

1. MovieS1 : Water droplets roll-off and bounce on superhydrophobic steel substrate.
2. MovieS2: Self-cleaning action of superhydrophobic steel substrate.
3. MovieS3: Glycerol drops roll-off on superoleophobic steel substrate.
4. MovieS4: Dodecane drop roll-off on superoleophobic steel substrate.
5. MovieS5: Self-healing ability of the Silicone oil infused slippery surfaces.


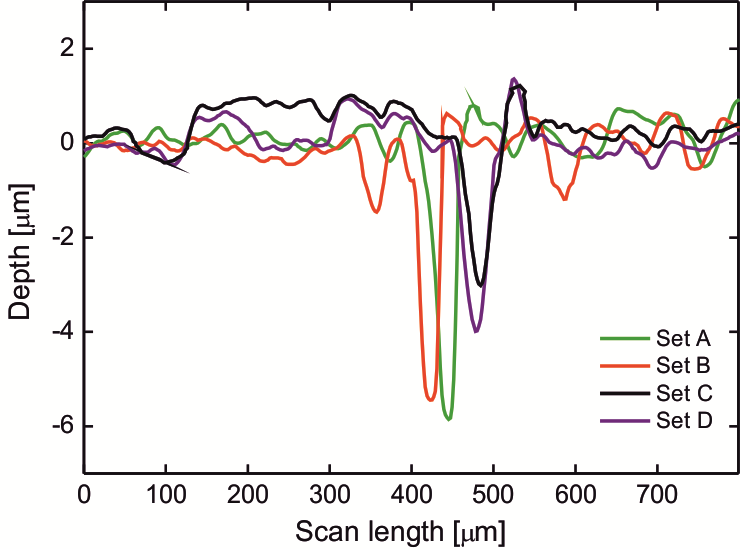


Figure S1: Thickness measurement of different CuO nanostructure coating obtained by mechanical profilometer.


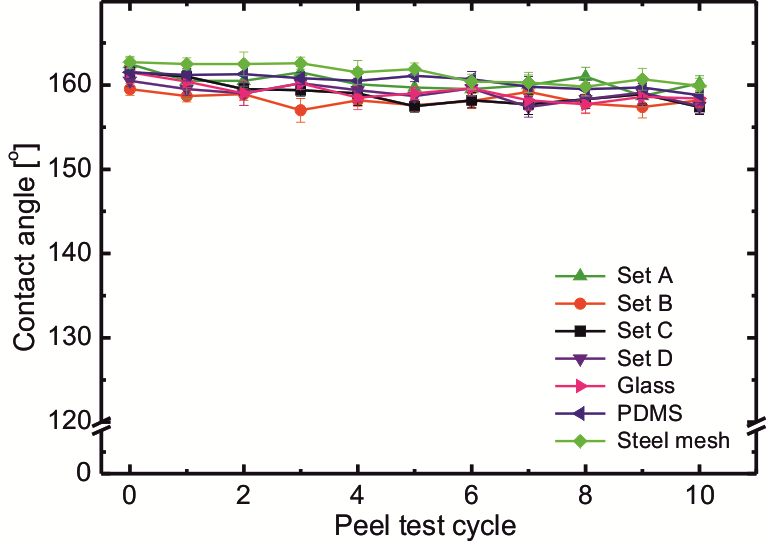


Figure S2. Mechanical stability test using tape test of the fabricated superhydrophobic samples.
